# An Adaptive Three-Dimensional Self-Masking Strategy for the Micro-Fabrication of Quartz-MEMS with Out-of-Plane Vibration Units

**DOI:** 10.3390/mi16060609

**Published:** 2025-05-23

**Authors:** Yide Dong, Chunyan Yin, Guangbin Dou, Litao Sun

**Affiliations:** SEU-FEI Nano-Pico Center, Key Laboratory of MEMS of Ministry of Education, Southeast University, Nanjing 210096, China; 230208145@seu.edu.cn (Y.D.); yincy@seu.edu.cn (C.Y.)

**Keywords:** quartz crystal, quartz MEMS, micro-fabrication, vibration units, tuning fork

## Abstract

Quartz crystal out-of-plane vibration units are critical components of QMEMS devices. However, the fabrication of their 3D sidewall electrode structures presents significant challenges, particularly within ultrafine etched grooves. These challenges seriously limit further miniaturization, which is critical for portable and wearable electronic applications. In this paper, we propose a novel 3D self-masking fabrication strategy that enables the precise formation of sidewall electrodes by using the etched beam structure as a self-aligned pattern transfer medium. Based solely on photolithography and wet etching processes, this approach overcomes the limitations of the conventional shadow mask technique by improving alignment accuracy, process efficiency, and fabrication yields. In addition, a predictive mathematical model was developed to guide process optimization, enabling adaptive and reliable fabrication. Sidewall electrodes were successfully achieved in etched grooves as narrow as 45 μm, closely matching the theoretical predictions. To validate the approach, an ultra-miniaturized out-of-plane vibration unit with a beam spacing of just 150 μm—the narrowest reported to date—was fabricated, representing an 80% reduction compared to previously documented structures. The unit exhibited a repeatability error below 1.13%, confirming the precision and reliability of the proposed fabrication strategy.

## 1. Introduction

**Figure 1:**
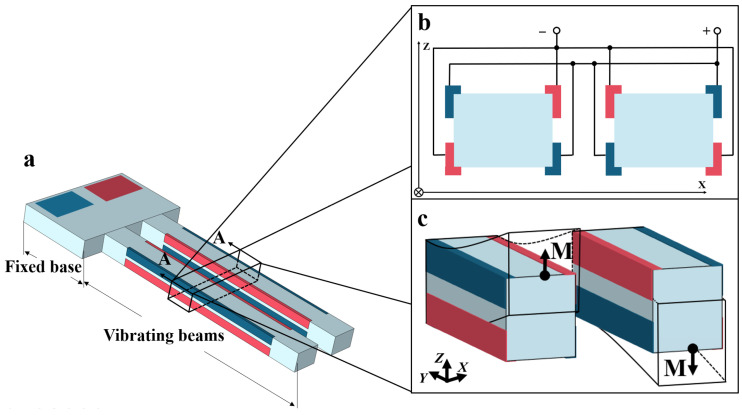
(**a**) Schematic diagram of a typical quartz crystal out-of-plane vibration unit; (**b**) layout of the sidewall discrete electrodes; (**c**) vibration modes of the out-of-plane vibration units.

Quartz crystal out-of-plane vibration units have become one of the most widely used and indispensable quartz devices, with applications in gyroscopes [1,2,3], pressure sensors [4,5,6], quartz-enhanced photoacoustic spectroscopy [7,8,9], and on-contact probes [10,11,12]. Figure 1 illustrates the schematic of a typical quartz crystal out-of-plane vibration unit, which consists of a fixed base and two vibrating beams. Notably, the 3D sidewall discrete electrodes are essential structures. Figure 1b shows the layout of the sidewall discrete electrodes on the vibrating beams. When electric fields of opposite polarity are applied to the top and bottom surfaces of the vibrating beams, polarized electric fields are induced along the X-plane of the quartz crystal, generating tensile and compressive stresses in opposite direction along the Y-axis. This stress distribution forces the beams to produce out-of-plane vibrations along the Z-axis, as illustrated in Figure 1c.

**Figure 2:**
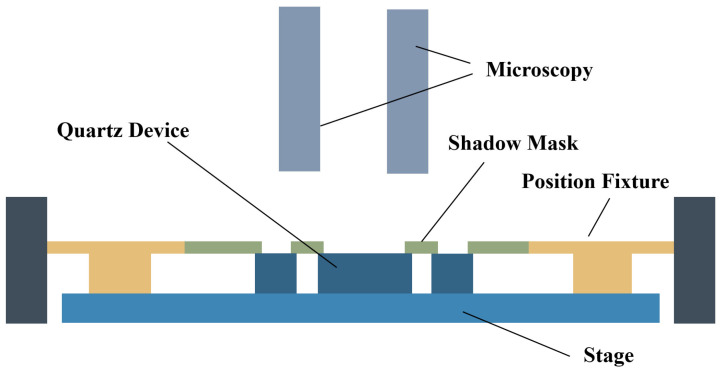
Schematic of a shadow mask alignment system.

**Figure 3:**
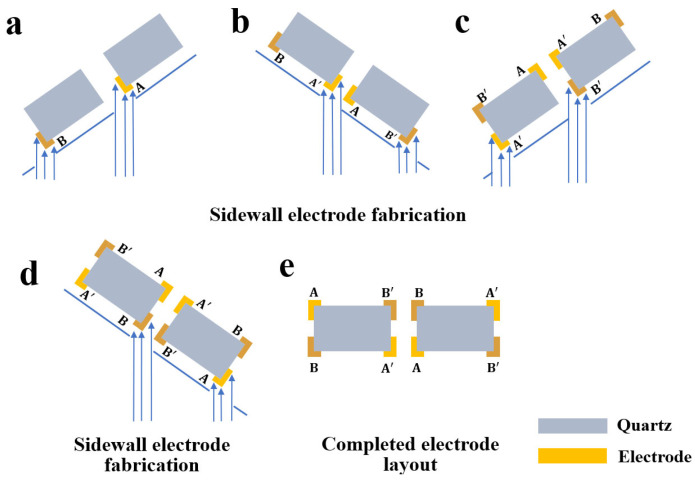
Process flow diagrams for the shadow mask method for depositing sidewall electrodes.

Currently, such 3D sidewall discrete electrodes are typically fabricated using shadow mask and evaporation techniques [2,13,14]. Figure 2 illustrates the equipment used for shadow mask alignment. As shown, the quartz crystal wafer is positioned on a fixed base. The shadow mask, designed with apertures to the layout of the evaporation electrodes, is placed tightly against the surface of the quartz wafer. Both the wafer and the mask are pre-aligned using an external fixture. The relative positions are in-tuned manually through a microscope, ensuring perfect alignment between the quartz wafer and the hard mask. Since the alignment is confirmed, an external fixing peg is used to secure the entire alignment system. The wafer, now covered with the stainless-steel shadow mask, is then placed into metal evaporation deposition equipment to fabricate the sidewall electrodes at an angle. The high collimation provided by the vacuum evaporation process ensures precision during deposition. These steps are repeated for both the front electrodes and the 3D sidewall electrodes. The flow of the deposition process is shown in Figure 3.

It is clear that the method using shadow masks for fabricating sidewall electrodes presents three main challenges: it is a cumbersome process, it has poor alignment accuracy, and there are limitations in terms of miniaturization.

Firstly, a total of four coating metal film operations are required to fabricate the face and sidewall electrodes, with the sidewall electrodes alone necessitating four coatings, resulting in an extremely complex and costly process. Secondly, due to positioning errors inherent in the shadow mask alignment equipment, cumulative alignment errors tend to increase. Shadow mask techniques demand very high alignment precision. In particular, even slight misalignments can cause a short-circuit between sidewall electrodes in ultrafine devices. And the use of shadow masks for electrodes can also lead to electrode vignetting, which affects the accuracy of the electrodes. Currently, there is no automation solution for this process, meaning all alignment must be performed manually, leading to low efficiency and yield. Moreover, the minimum processing tolerance for conventional stainless-steel shadow masks is ±20 μm, which necessitates a spacing of more than 1 mm between the two vibration beams to ensure a safer and higher-yield 3D sidewall electrode coating process. This severely restricts the miniaturization of out-of-plane vibration units. For example, the ST8 model produced by BEI achieved device dimensions of 11.2 × 2.4 × 0.25 mm^3^, the smallest device width reported to date [15], and it has nearly reached the theoretical limit for the shadow mask process. In addition, laser fabrication [16] or methods involving building external structures [17,18] have been used to fabricate sidewall electrodes for other substrate materials. However, all of these methods are only suitable for fabricating sidewall electrodes on protruding structures rather than in high-aspect-ratio etching grooves. At the same time, with the growing demand for wearable and portable devices, the need for further device miniaturization is becoming increasingly urgent. The current shadow-mask-based fabrication method of 3D sidewall discrete electrodes has become a significant bottleneck. Thus, the development of novel 3D processing is urgently required.

In this paper, a QMEMS adaptive 3D mask sidewall electrode processing strategy is proposed, enabling the fabrication of 3D sidewall electrodes within ultrafine etching grooves by utilizing the etched beam structure as a pattern transfer medium for sidewall discrete electrodes. The experimental results demonstrated show that this all-photolithography process effectively addressed the issues of low alignment accuracy, low processing efficiency, low product yield, and limited miniaturization associated with traditional shadow mask methods. Notably, a quantitative predictive mathematical model was established, correlating the effective width of the sidewall electrode with the hard mask width and etching time, thereby enabling adaptive and predictable fabrication results. Using this approach, sidewall electrodes were successfully fabricated within etched grooves as narrow as 45 μm, consistent with theoretical predictions. Furthermore, the proposed strategy was employed to fabricate an ultra-miniaturized out-of-plane vibration unit featuring a beam spacing of only 150 μm, the narrowest reported to date, representing an 80% reduction compared to previously documented structures. The fabrication process achieved a repeatability error of less than 1.2%, demonstrating its high precision and reliability. These results confirm that the self-masking fabrication strategy significantly reduced the dimensions of such vibration units, offering strong potential for application in wearable and mobile devices.

## 2. Materials and Methods

### 2.1. Pattern Design for Narrow Strips

**Figure 4:**
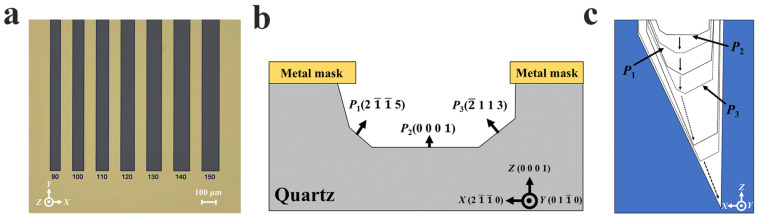
(**a**) Partial pattern designs used in this work; (**b**) definition of bilateral etching profile; (**c**) evolution of the complete etching profile during wet etching of Z-cut quartz crystal.

To address the miniaturization requirement of QMEMS devices, this study designed a series of ultra-narrow hard mask patterns to investigate the etching morphology evolution of quartz crystal. The initial long-strip patterns had a width ranging from 10 μm to 150 μm (Figure 4a). The strips measured 1 mm in length, were spaced 500 μm apart, and were aligned along the Y-direction.

### 2.2. Sample Preparation

Z-cut quartz crystal wafers with a thickness of 120 μm were used in this study. The wafer, supplied by Crystron Technologies Inc. (Nanjing, China), met the quality requirements of a Q value ≥ 2.4 million and an etched channel density ≤ 30 bars/cm^2^. Prior to processing, the quartz wafers were cleaned in piranha solution (H_2_SO_4_:H_2_O_2_ = 3:1) at 90 °C for 20 min. A set of Au (310 nm) and Cr (60 nm) films was sputtered onto the quartz wafers as hard mask layers using a Kurt J. Lesker PVD 75 system (Jefferson Hills, PA 15025 USA). The hard mask layers were subsequently patterned using the designed photoresist mask. Then, etching was performed in a mixed etchant composed of 49 wt. % HF and 40% wt. % NH_4_F (HF:NH_4_F = 2:3) at 80 ± 1 °C. Samples were removed from the etchant every 10 min over a total etching time of 120 min. To minimize random errors, three samples were etched simultaneously, and the etching depth of each sample was measured separately.

### 2.3. Characterization of Bilateral Etching Profiles

In this study, a novel and concise definition is proposed for characterizing small-hard-mask-width etching profiles. As shown in Figure 4b, the complete bilateral etched groove is demonstrated, differing from the previous work where only half of the etched profile was defined. Furthermore, angles were not included in the definition, simplifying the characterization of the etching profile. Instead, three planes—**P_1_**$\left(2\bar{1}\bar{1}5\right)$, **P_2_**(0001), and **P_3_**$\left(\bar{2}113\right)$—are defined to fabricate the presentation of the etching profile evolution. Figure 4c illustrates this evolution in an aggregated manner. The completed etching evolution was discussed in our earlier work [19]; however, a brief summary is provided here for clarity: As the etching depth increases, the lengths of **P_1_** and **P_3_** extend while the **P_2_** platform first shrinks and eventually disappears. Thereafter, **P_1_** and **P_3_** gradually shrink and vanish sequentially. Finally, after **P_3_** disappears, an asymmetric sharp etching groove is formed.

### 2.4. Etching Profile and Device Characterization

After etching, the quartz wafers were cut perpendicular to the etching slots, and the resulting etching morphologies were observed and measured using optical microscopy (Olympus BX53M, Suzhou, China) and a surface profiler (Dektak, Billerica, MA, USA). The fabricated vibration units were measured and subsequently characterized in terms of their electrical parameters using an impedance analyzer (Tonghui TH2838, Suzhou, China).

## 3. Results and Discussion

### 3.1. Fundamental Principle of Self-Masking Micro-Fabrication Strategy

**Figure 5:**
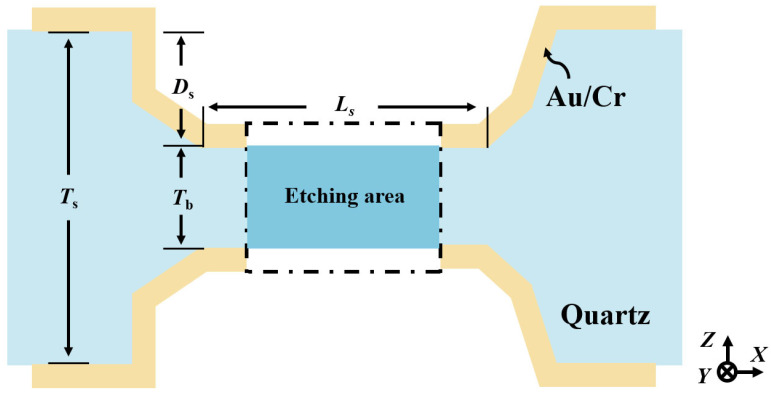
Schematic diagram of the adaptive 3D self-masking micro-fabrication strategy.

The core principle of the QMEMS adaptive 3D mask sidewall electrode fabrication strategy is to use the beam structure formed by quartz etching as the mask layer of the discrete sidewall electrodes, enabling their fabrication through secondary coating, photolithographic patterning, and the wet etching process. Figure 5 illustrates a schematic diagram of the adaptive 3D mask sidewall electrode fabrication strategy.

For clarity in characterizing the etching topography, the length definitions are induced: the length of the bottom of the etching groove (i.e., the 3D self-masking beam) is denoted by $L_{s}$; $T_{b}$ and $T_{b}$ represent the thickness and length of the junction between the 3D mask beam and the vibration beams, respectively; $T_{s}$ corresponds to the wafer thickness; and $D_{s}$ indicates the etching depth. In practical application, an appropriate initial hard mask width is selected based on the wafer thickness and the etching evolution results. After the etching grooves are patterned, a double-sided etching process is performed to form an H-type beam with a controlled thickness. At this stage, both ends of the mask beam remain connected to the vibration beams. Due to the presence of the junction thickness $T_{b}$, the mask beam can serve as a 3D sidewall electrode mask. Subsequently, a metal film is deposited again onto the substrate via deposition for sputtering to form a secondary hard mask layer. After patterning, the metal layer on the self-masking beam is selectively removed, and both the vibration beams and mutually discrete 3D sidewall electrodes are simultaneously obtained.

It is evident that this eliminates shadow mask and mechanical alignment structures. Alignment relies solely on the mark point patterned onto the wafer, meaning the lithography accuracy directly determines the alignment accuracy of the sidewall electrodes. As a result, the achievable alignment precision is significantly higher than that of the shadow mask method. Typically, lithography offers at least one order of magnitude greater alignment accuracy compared to shadow mask techniques, while also providing superior theoretical resolution limits, thereby greatly enhancing fabrication precision. Furthermore, this strategy removes the need for complicated manual alignment and the fixture assembly process, thereby improving production efficiency and yield. In addition, by eliminating the limitations imposed by the stainless-steel shadow mask’s processing tolerances, this method enables the fabrication of 3D sidewall electrodes within the ultra-high-aspect-ratio etching grooves. By employing a finely controlled hard mask width with innovative undercut processes, the obstacles to miniaturizing the out-of-plane vibration unit are effectively overcome. Finally, the size of the resulting sidewall electrodes can be adaptively controlled by adjusting the etching depth of the self-masking beam.

**Figure 6:**
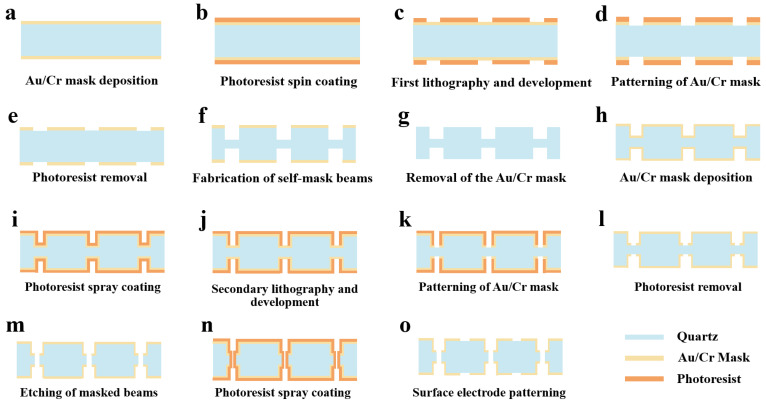
Process flow diagram of the controllable 3D self-masking micro-fabrication strategy.

Figure 6 shows the entire process flow of the QMEMS adaptive 3D mask sidewall electrode fabrication strategy. Initially, the wafer surface is cleaned with strong acids and organic solvents. After surface treatment, an Au/Cr mask layer is deposited on both sides of the wafers using vapor deposition or magnetron sputtering (Figure 6a). Next, a photoresist layer is spin-coated onto the wafer (Figure 6b), followed by the first round of double-sided photolithography and development (Figure 6c). The metal hard mask layer is patterned (Figure 6d), and the photoresist is removed (Figure 6e). The wafer is immerged in an etching solution to make the self-masking beam. The corresponding etching depth is selected based on the design of the sidewall electrodes (Figure 6f). Subsequently, the metal mask layer is removed (Figure 6g). After washing, the metal mask layer is re-deposited for the secondary etching process (Figure 6h). A new photoresist is sprayed onto the wafer (Figure 6i), followed by secondary photolithography and development (Figure 6j). The secondary metal layer is etched to pattern the metal mask (Figure 6k), and the photoresist is stripped (Figure 6l). The wafer is then placed in the etching solution once more to remove the self-masking beam through secondary etching. In this process, the Au/Cr film acts as the hard mask on the quartz substrate (Figure 6m). The wafer is coated with photoresist again (Figure 6m) and the surface electrodes are patterned (Figure 6n). Finally, the photoresist is stripped and the wafer cleaned (Figure 6o).

In this way, a beam with front electrodes and 3D discrete sidewall electrodes is fabricated. At this point, the metal layer used for the hard mask also serves as the electrode layer. This novel strategy significantly reduces the complexity of the sidewall electrode fabrication process and greatly lowers production cost. The entire process is realized through photolithographic alignment, ensuring high-precision processing for ultra-small devices.

**Figure 7:**
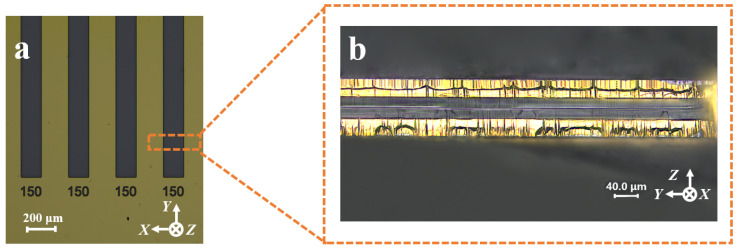
(**a**) Initial hard mask pattern with a width of 150 μm; (**b**) fabricated sidewall discrete electrodes with etched grooves of 150 μm.

To verify the feasibility of the QMEMS adaptive 3D mask sidewall electrode fabrication process and to enhance its value for practical engineering applications, sidewall discrete electrodes were fabricated within an etching groove with an initial hard mask width of 150 μm following the process flow outlined in Figure 6. The sample preparation and etching procedures are detailed in Section 2. The results of the etching process are presented in Figure 7. It was evident that 3D sidewall discrete electrodes were successfully fabricated within such fine etched grooves. To further confirm the effectiveness of the proposed strategy, the continuity between the two sidewall electrodes was tested using a probe station and a multimeter. The measurement results demonstrated that the two sidewall electrodes were electrically isolated, thereby validating the feasibility of the adaptive 3D mask process.

### 3.2. Adaptive and Predictable Fabrication of 3D Sidewall Electrodes

The dimensional characteristics of 3D sidewall electrodes have a critical impact on the performance of quartz-based devices. Conventional fabrication methods employing shadow masks are unable to achieve precise and adaptive control of electrode dimensions. In contrast, the self-masking fabrication strategy enables the realization of sidewall electrodes with adaptive and predictable dimensions, as the final electrode size can be accurately tuned based on the etching depth during the initial process step.

**Figure 8:**
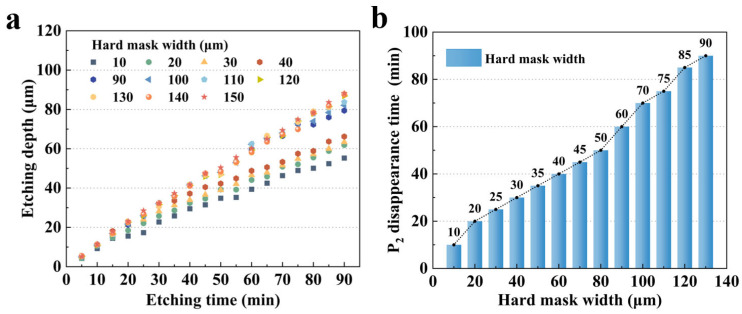
(**a**) Influence of initial mask width on etching depth during quartz crystal etching; (**b**) relationship between **P_2_** plane disappearance time and initial hard mask width.

However, due to the size effect in the wet etching of quartz crystal [19,20], different initial hard mask widths result in varying etch depths. As shown in Figure 8a, it is evident that a wider initial hard mask leads to a deeper etching depth with the same etching time. At 90 min, the difference in etch depth exceeded 20 μm. In addition, this size effect also significantly influenced the etching morphology. Figure 8b illustrates the variation in the **P_2_** platform disappearance time with respect to different initial metal mask widths. It can be observed that the disappearance time of the **P_2_** plane increased almost linearly with the initial mask width. Specifically, the **P_2_** disappearance time of a 130 μm mask was nearly 80 min longer than that of a 10 μm mask. These factors collectively impact the final morphology of the sidewall electrodes.

**Figure 9:**
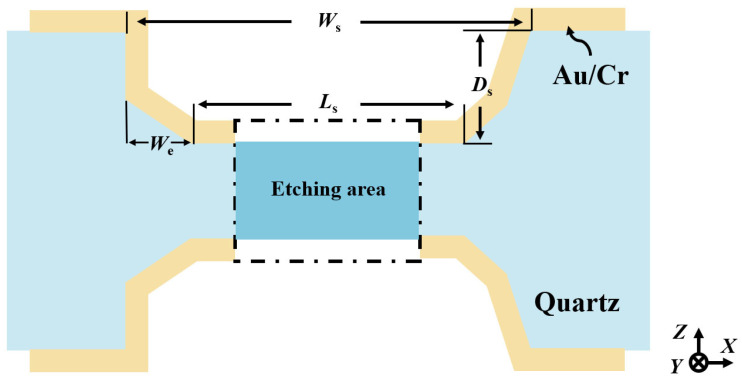
Simplified schematic of the etched cross-sectional structure.

Therefore, to realize an adaptive self-masking fabrication strategy, it is crucial to establish a predicative mathematical model based on extensive etching data analysis. To facilitate this analysis, a simplified schematic of the etched cross-section is provided in Figure 9. Here, $W_{s}$ denotes the etching groove width, $D_{s}$ represents the etching depth and the effective width of the sidewall electrodes, and $L_{s}$ corresponds to the length of the self-masking beam.

Based on the cross-sectional definition, the actual etching depth $D_{s}$ can be equated to the effective electrode width of the sidewall electrodes. Therefore, according to the size effect, the effective width $D_{s}$ is jointly determined by the initial hard mask width $W_{s}$ and the etching time t. To predict the fabrication dimensions of the sidewall electrodes, $D_{s}$ is defined in this work as a function of $W_{s}$ and t, as expressed in Equation (1):

(1) $$D_{S}=f\left(W_{s},t\right)$$

**Figure 10:**
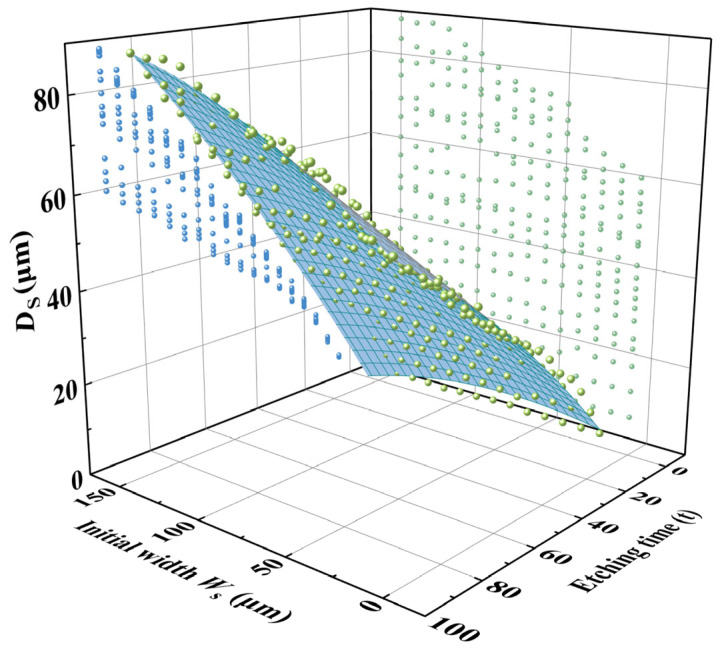
Three-dimensional plot of etching depth $D_{s}$ for initial hard mask widths ranging from 10 μm to 150 μm.

The etching results of initial hard mask widths ranging from 10 μm to 150 μm were plotted into a three-dimensional coordinate system, as shown in Figure 10.

It was evident that $D_{s}$ is influenced by both the etching time and the initial hard mask width. Particularly for small initial hard mask widths, the growth of $D_{s}$ slowed down markedly with increasing etching time due to the pronounced size effect. In contrast, the impact of size effect on $D_{s}$ is less significant.

The relationship of $D_{s}$ was obtained by fitting the experimental data using a binary quadratic (Poly2D) function, leading to the effective width prediction model shown in Equation (2):

(2) $$D_{s}=Z_{0}+Ax+By+Cx^{2}+Dy^{2}+Fxy$$

where the **x** is the etching groove width $W_{s}$ and **y** means the etching time t.

The experimental data were substituted in Equation (2) for fitting, and the resulting coefficients along with their coefficients of determination ($R^{2}$) are summarized in Table 1.

The table shows that the coefficient of determination, $R^{2}$, was as high as 0.994, indicting a strong fit of the coefficients. The fitting function accurately represents the change of etching depth under the combined influence of initial hard mask width and etching time. Based on this equation, it is possible to predict the effective electrode width, $D_{s}$, at any etching time for any initial hard mask width, enabling precise control over the 3D sidewall discrete electrodes.

### 3.3. Fabrication of 3D Sidewall Electrodes in Ultrafine Etching Grooves

In this section, the process limitations of the adaptive 3D self-masking fabrication strategy are discussed.

For practical application, to ensure both feasibility and repeatability, the self-masking beam length $L_{S}$ was selected as the lithographic hard mask pattern width for secondary lithography. Due to size effects, the etching platform width $L_{S}$ is jointly influenced by the initial hard mask width $W_{S}$ and the etching time *t*.

Therefore, $L_{S}$ can be defined as a binary function of $W_{S}$ and *t*, as expressed in Equation (3):

(3) $$L_{S}=f\left(W_{s},t\right)$$

**Figure 11:**
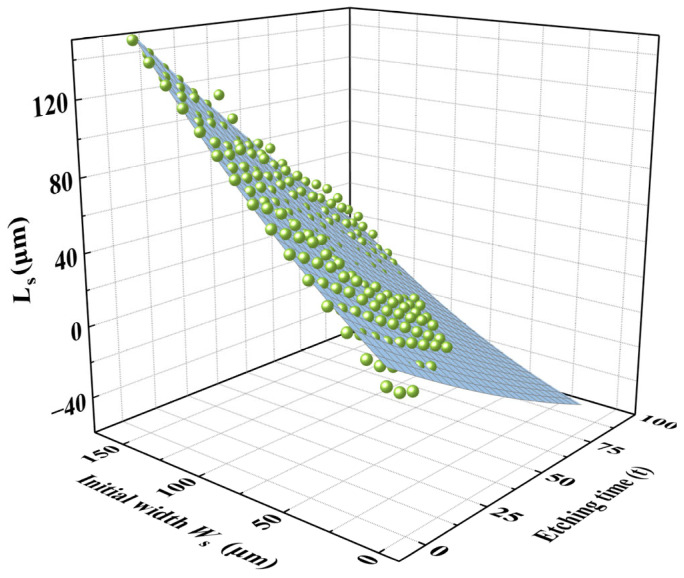
Three-dimensional plot of the etching platform with $L_{S}$ being a function of initial hard mask widths ranging from 10 μm to 150 μm.

The etching results for initial hard mask widths ranging from 10 μm to 150 μm were plotted in a 3D coordinate system, as shown in Figure 11.

Similarly, the size effect on $L_{S}$ was negligible when the etching time was short or when the initial hard mask width was relatively large. However, for smaller initial hard mask widths combined with longer etching times, the size effect on $L_{S}$ became increasingly significant.

Since $L_{S}\;$ is also a binary function of the initial hard mask width and etching time, an empirical relationship can be established by fitting the experimental data using a binary quadratic (Poly2D) function. Thus, $L_{S}\;$ could be expressed as shown in Equation (4):

(4) $$L_{S}=Z_{0}+Ax+By+Cx^{2}+Dy^{2}+Fxy$$

where the **x** is the etching platform width $L_{S}$ and **y** means the etching time t.

The experimental data were substituted in Equation (4) and fitted accordingly. The resulting coefficients, along with their corresponding coefficients of determination ($R^{2}$), are summarized in Table 2.

The data in Table 2 show that the coefficient of determination *R^2^* reached 0.989, demonstrating a strong fit of the coefficients.

To ensure that the sidewall discrete electrodes remain electrically isolated, it is necessary to maintain the presence of the etching self-masking beam even when the etching groove reaches approximately half of the wafer thickness.

In practice, extending the secondary etching time can flatten the sidewall rise, thereby improving device performance [21]. However, excessive etching may cause discrete electrodes to short-circuit. Thus, a certain degree of design redundancy must be incorporated into the process to prevent the sidewall from leading to electrode short-circuiting, thereby enhancing the stability and reproducibility of the technology.

**Figure 12:**
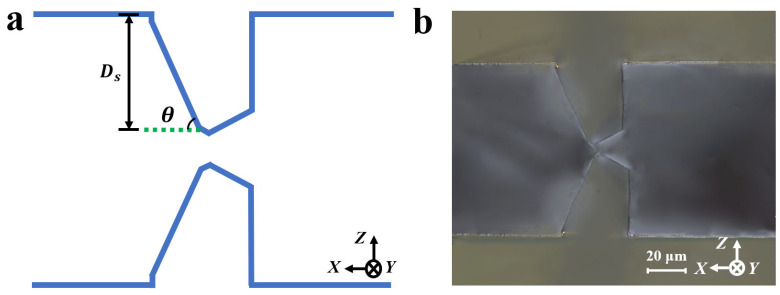
(**a**) Schematic cross-sectional etching morphology; (**b**) etching cross-section of initial hard mask with a width of 35 μm after 60 min of etching.

To avoid electrode short-circuiting, it is essential to satisfy the conditions shown in Equation (5), as illustrated in Figure 12:

(5) $$\frac{D_{s}}{\sin\theta }<\frac{T}{2}$$

where T is the wafer thickness and θ is the angle between the etched crystal plane and the Z-direction.

In this work, T = 120 μm, θ = 61°, yielding a maximum allowable $D_{s}$ of 52.4 μm. For simplicity in subsequent calculations, $D_{s}$ was set to 52 μm.

In summary, $L_{s}$ must satisfy the condition expressed in Equation (6):

(6) $$L_{S}=f\left(W_{s},t_{52}\right)>0$$

where $t_{52}\;$ is the etching time when $D_{s}=52\;\mu m$. This can be expressed as follows, according to Equation (7):

(7) $$t=f^{\prime }\left(W_{s},D_{s}\right)$$

Thus, for $t_{52}$, we have the following:

(8) $$t_{52}=f^{\prime }\left(W_{s},52\right)$$

To determine the thinnest initial hard width, $W_{min}$, that can be manufactured under this condition, $W_{min}$ must satisfy Equation (9):

(9) $$L_{S}=f\left(W_{min},f^{\prime }\left(W_{min},52\right)\right)=0$$

Substituting the coefficients obtained from Table 2 into the $L_{s}$ function, the expression for $L_{s}$ is as follows:

(10) $$L_{s}=12.29+0.691x-0.945y-\left(1.65\times 10^{-3}\right)x^{2}+\left(1.92\times 10^{-3}\right)y^{2}-\left(4.61\times 10^{-3}\right)xy$$

For the x-term coefficients, given that

(11) $$0.691\gg (1.65\times 10^{-3})x$$

and

(12) $$0.691\gg (4.61\times 10^{-3})y$$

we can neglect the terms $\left(1.65\times 10^{-3}\right)x^{2}$ and $\left(4.61\times 10^{-3}\right)xy$.

Similarly, for *y*-term coefficients,

(13) $$0.945\gg \left(1.92\times 10^{-3}\right)y$$

so we can disregard $\left(1.92\times 10^{-3}\right)y^{2}$.

Thus, $L_{s}\;$ could be simplified as follows:

(14) $$L_{s}=12.29+0.691x-0.945y$$

Substituting the coefficients into the $D_{s}$ function, we obtain

(15) $$D_{s}=1.12+\left(8.4\times 10^{-2}\right)x+0.8y+\left(7.8\times 10^{-4}\right)x^{2}+\left(2.8\times 10^{-3}\right)xy$$

Now, $f^{\prime }(W_{min},52)$ can be calculated as follows:

(16) $$f^{\prime }\left(W_{min},52\right)=\frac{48.88-\left(8.4\times 10^{-2}\right)W_{min}-\left(7.8\times 10^{-4}\right){W_{min}}^{2}}{0.8+\left(2.8\times 10^{-3}\right)W_{min}}$$

Setting $f\left(W_{min},f^{\prime }(W_{min},52)\right)=0$, the following equation can be solved:

(17) $$\frac{48.88-\left(8.4\times 10^{-2}\right)W_{min}-\left(7.8\times 10^{-4}\right){W_{min}}^{2}}{0.8+\left(2.8\times 10^{-3}\right)W_{min}}=\frac{12.29+0.69W_{min}}{0.945}$$

**Figure 13:**
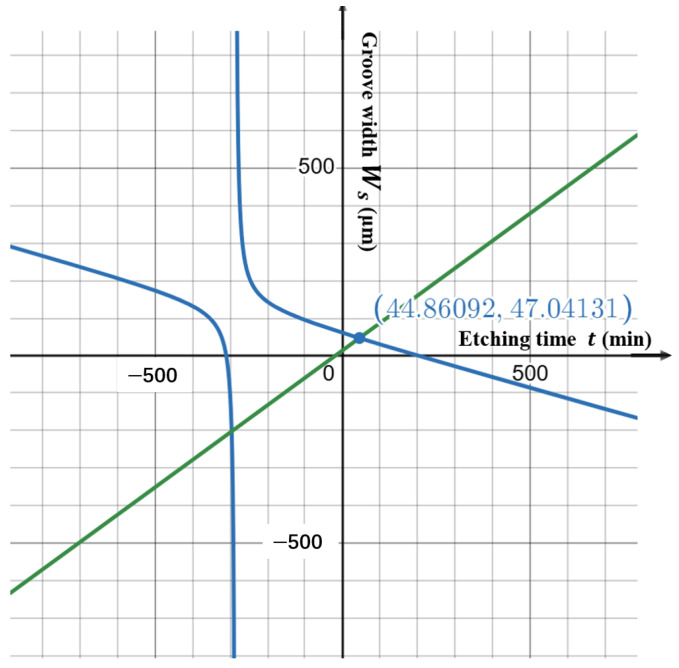
Graphical solution for determining the minimum manufacturable initial hard mask width.

The graph of the above equation is shown in Figure 13. By using the graphical solutions, the minimum initial hard mask width $W_{min}=44.8\;\mu m$ was determined for this process condition.

**Figure 14:**
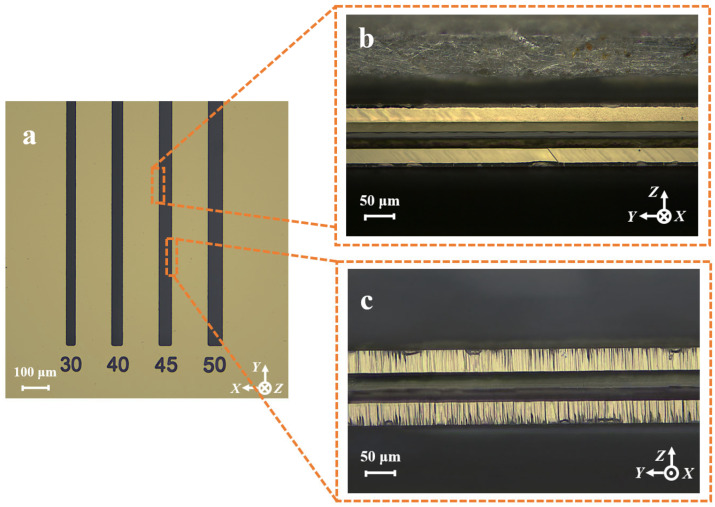
(**a**) Etching groove with an initial hard mask width of 45 μm; (**b**) +X-plane sidewall discrete electrode; (**c**) −X-plane sidewall discrete electrode.

To validate the theoretical calculations, sidewall discrete electrodes were fabricated using the 3D adaptive self-masking micro-fabrication strategy in a deep etching groove with an initial hard mask width of 45 μm. The fabrication results are shown in Figure 14. It is evident that 3D sidewall electrodes were successfully formed on both the +X side (Figure 14b) and the −X side (Figure 14c). This strategy enables the fabrication of 3D sidewall electrodes within ultrafine etching grooves, providing a pathway toward the extreme miniaturization of QMEMS out-of-plane vibration units.

### 3.4. Fabrication of Ultra-Small Vibration Unit Based on Self-Masking Fabrication Strategy

**Figure 15:**
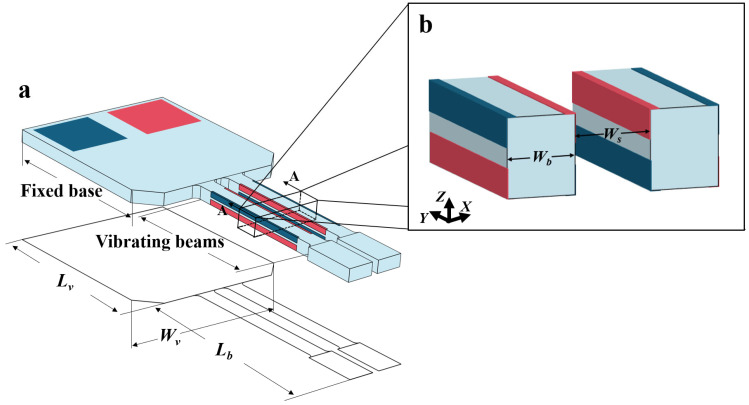
(**a**) Overall design of the out-of-plane vibration unit; (**b**) layout of sidewall electrodes.

The self-masking etching strategy was employed to fabricate an ultra-small out-of-plane vibration unit on a 120 μm thick quartz wafer. Figure 15 illustrates the structure and key dimensions of the unit. The vibration unit consists of vibration beams and a supporting base. Each vibration beam has a length of 2000 μm and a width of 175 μm, and the gap between two adjacent beams $W_{s}$ is only 150 μm. The key dimensional parameters of the unit are summarized in Table 3.

**Figure 16:**
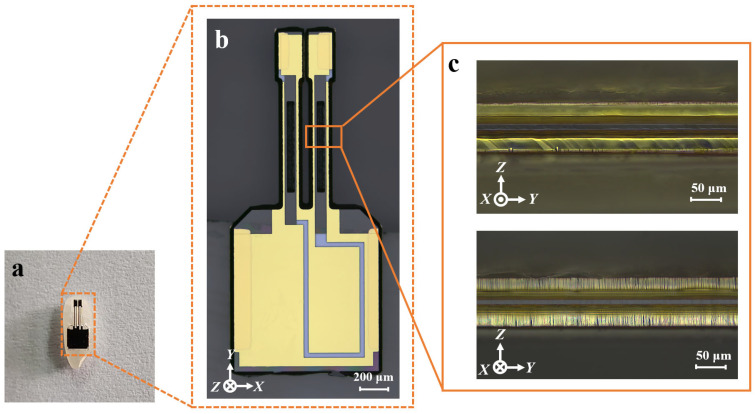
(**a**) Vibration unit placed on a rice grain; (**b**) the vibration unit; (**c**) vibration beam in the x-direction with sidewall electrodes.

The vibration unit was fabricated by using the self-masking strategy. Figure 16 presents a cross-sectional view of the completed vibrating unit and its vibrating beam. To provide a sense of scale, the unit is shown placed on a grain of rice (Figure 16a). As seen from the cross-section image (Figure 16c), the sidewalls exhibit uniform discrete electrodes.

**Figure 17:**
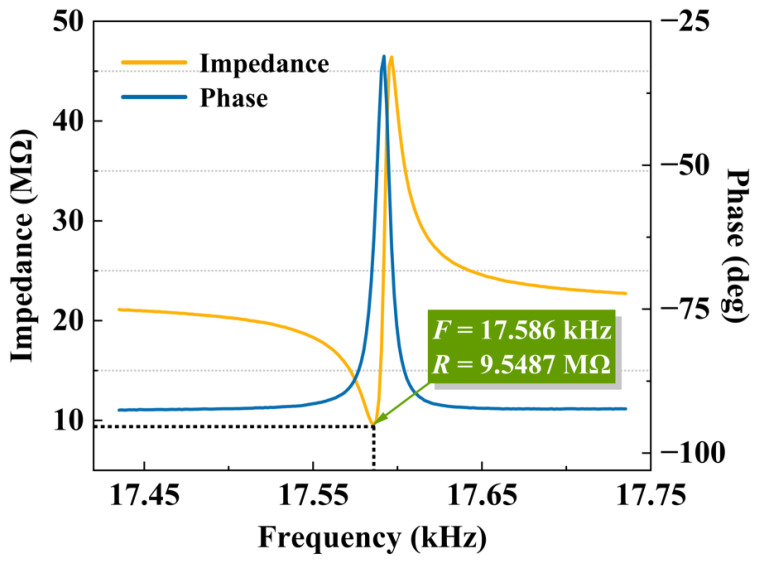
Test result of the vibration unit.

The vibration unit was tested using an impedance analyzer at normal atmospheric pressure, and the test results are shown in Figure 17. The resonant frequency was measured at 17.58 kHz, with a resonant impedance of 9.55 MΩ, both of which align with the design specifications.

In this study, five units were fabricated and tested. The statistical results are presented in Table 4. The repeatability error is defined as follows:

(18) $$E=\frac{\sigma }{\left|\bar{x}\right|}\times 100\%$$

where σ is the standard deviation of multiple measurements, and $\bar{x}$ is the absolute value of the average of multiple measurements.

The repeatability errors in a wafer were also tested, as presented in Table 5.

As shown in the table, the process repeatability errors for the critical dimensions of the device were all within 1.13% and the repeatability errors in a wafer were all within 0.74%, demonstrating that the innovative fabrication method proposed in this paper exhibits excellent repeatability. This indicates that the self-masking fabrication strategy holds significant potential for large-scale mass production.

## 4. Conclusions

This paper proposes an adaptive 3D self-masking sidewall electrode processing strategy which enables the fabrication of 3D sidewall electrodes in ultrafine etched grooves. By utilizing the etched beam structure as a pattern transfer medium for the sidewall discrete electrodes, the strategy effectively addresses key challenges in traditional QMEMS processes, including low alignment accuracy, inefficient efficiency, low product yield, and limited miniaturization. Based on the etching data, a mathematical model was developed to predict the effective width of the 3D sidewall electrode as a function of the etching conditions, enabling adaptive and predictable electrode processing. Furthermore, sidewall electrodes were successfully fabricated in an etched groove as narrow as 45 μm, consistent with the calculated results. The proposed strategy was also applied to fabricate an ultra-miniaturized all-out-of-plane vibration unit with a resonant frequency of 17.58 kHz. This unit features the smallest reported vibration beam spacing, measuring only 150 μm—an 80% reduction compared to previously documented structures. The process demonstrated a repeatability error of less than 1.13%, confirming the viability of the strategy for overcoming the miniaturization bottleneck in out-of-plane vibration units. These results highlight the potential for the strategy to be implemented in wearable and mobile devices.

## Figures and Tables

**Table 1:** Coefficients from fitting $D_{S}$ results.

Coefficient	$Z_{0}$	A	B	C	D	F	$R^{2}$
Value	1.117	0.084	0.797	−7.781 × 10^−4^	−2.12 × 10^−3^	2.77 × 10^−3^	0.99394

**Table 2:** Fitting results for the coefficients of $L_{S}$.

Coefficient	$Z_{0}$	A	B	C	D	F	$R^{2}$
Value	12.29	0.691	−0.945	1.65 × 10^−3^	1.92 × 10^−3^	−4.61 × 10^−3^	0.98901

**Table 3:** Key dimensions of the vibration unit.

**Item**	**Size (μm)**	**Item**	**Size (μm)**
$\mathrm{Device}\;\mathrm{length}\;L_{v}$	3800	$\mathrm{Device}\;\mathrm{width}\;W_{v}$	1620
$\mathrm{Beam}\;\mathrm{length}\;L_{b}$	1970	$\mathrm{Beam}\;\mathrm{width}\;W_{b}$	185
$\mathrm{Space}\;W_{s}$	150	Thickness T	120

**Table 4:** The repeatability error of 5 units fabricated using the self-masking fabrication strategy.

Item	$\mathrm{Beam}\;\mathrm{Length}\;L_{b}$	$\mathrm{Beam}\;\mathrm{Width}\;W_{b}$	Frequency (kHz)
1	1970	185	17.586
2	1970	187	17.849
3	1971	182	17.266
4	1970	187	17.758
5	1971	186	17.592
σ	0.45	1.85	0.19
E	0.02%	1.00%	1.13%

**Table 5:** The repeatability errors in a wafer when using the self-masking fabrication strategy.

Item	Position	$\mathrm{Beam}\;\mathrm{Length}\;L_{b}$	$\mathrm{Beam}\;\mathrm{Width}\;W_{b}$	Frequency (kHz)
1	Top-left corner	1970	183	17.395
2	Lower-left corner	1969	187	17.794
3	Center	1970	184	17.491
4	Center	1971	185	17.568
5	Top-right corner	1971	185	17.568
6	Lower-right corner	1971	187	17.758
σ	/	0.69	1.35	0.13
E	/	0.04%	0.73%	0.74%

## Data Availability

All data generated or used during the study are included in the submitted article.
